# The paradox of cooperation among selfish cancer cells

**DOI:** 10.1111/eva.13571

**Published:** 2023-07-07

**Authors:** Jean‐Pascal Capp, Frédéric Thomas, Andriy Marusyk, Antoine M. Dujon, Sophie Tissot, Robert Gatenby, Benjamin Roche, Beata Ujvari, James DeGregori, Joel S. Brown, Aurora M. Nedelcu

**Affiliations:** ^1^ Toulouse Biotechnology Institute University of Toulouse, INSA, CNRS, INRAE Toulouse France; ^2^ CREEC, MIVEGEC University of Montpellier, CNRS, IRD Montpellier France; ^3^ Department of Cancer Physiology H Lee Moffitt Cancer Center and Research Institute Tampa Florida USA; ^4^ Centre for Integrative Ecology, School of Life and Environmental Sciences Deakin University Geelong Victoria Australia; ^5^ Department of Biochemistry and Molecular Genetics University of Colorado Anschutz Medical Campus Aurora Colorado USA; ^6^ Department of Biology University of New Brunswick Fredericton New Brunswick Canada

**Keywords:** cancer, cheating, cooperation, greenbeard, selfishness

## Abstract

It is traditionally assumed that during cancer development, tumor cells abort their initially cooperative behavior (i.e., cheat) in favor of evolutionary strategies designed solely to enhance their own fitness (i.e., a “selfish” life style) at the expense of that of the multicellular organism. However, the growth and progress of solid tumors can also involve cooperation among these presumed selfish cells (which, by definition, should be noncooperative) and with stromal cells. The ultimate and proximate reasons behind this paradox are not fully understood. Here, in the light of current theories on the evolution of cooperation, we discuss the possible evolutionary mechanisms that could explain the apparent cooperative behaviors among selfish malignant cells. In addition to the most classical explanations for cooperation in cancer and in general (by‐product mutualism, kin selection, direct reciprocity, indirect reciprocity, network reciprocity, group selection), we propose the idea that “greenbeard” effects are relevant to explaining some cooperative behaviors in cancer. Also, we discuss the possibility that malignant cooperative cells express or co‐opt cooperative traits normally expressed by healthy cells. We provide examples where considerations of these processes could help understand tumorigenesis and metastasis and argue that this framework provides novel insights into cancer biology and potential strategies for cancer prevention and treatment.

## 1 INTRODUCTION

Multicellular individuals evolved from groups of previously independent single‐celled individuals, through either aggregation (such as in slime molds) or failure to separate after cell division (e.g., most multicellular lineages, including animals) (Grosberg & Strathmann, 2007). For multicellular groups to become stable units of evolution (evolutionary individuals) in their own right, variation and selection among cells within the group have to be controlled such that selection can act among groups (i.e., on the newly emerged multicellular individual) (Michod, 1997). However, cells in a multicellular individual can still acquire mutations and maintain the necessary conditions to evolve (heritable variation in fitness). Thus, variation can still occur, and selection can still act at the cell level. A series of processes have evolved to control intra‐organismal evolution through (i) reducing the incidence of mutations (limiting genetic variation) and (ii) lowering the negative effects of such mutations by decreasing their selective advantage (limiting cell‐level selection) (DeGregori, 2011). Cancer cells exemplify the failure of these mechanisms as they are characterized by increased genetic variation and fitness relative to the normal cells. Under specific circumstances, cell‐level selection can favor cancer cells to the detriment of the organism, ultimately resulting in its demise.

Within groups, interactions occur that can affect the fitness of group members. Based on their effect on the fitness of the actor and recipient, such interactions can be classified into cooperative (i.e., mutually beneficial or altruistic), selfish and spiteful (Gardner & West, 2010; West et al., 2007a). Similar interactions can also occur within groups of cells. The transition to multicellularity required the evolution of stable cooperative cell–cell interactions that increased the fitness of the group relative to other groups. These interactions—from mutually beneficial (as in the production of adhesion molecules) to altruistic (as in suppression of cell proliferation or activation of programmed cell death), are reflected in a series of adaptations at the group level that ensure the integration and coordination of all members toward improved functionality and fitness of the multicellular organism as a whole (Box 1).

BOX 1 **Cooperation in multicellular organisms.**

*Theirs not to make reply, Theirs not to reason why, Theirs but to do and die*.

*Into the valley of Death Rode the six hundred*.

(from “Charge of the Light Brigade,” Alfred Lord Tennyson)

In multicellular organisms, somatic cells fit Tennyson's poetry. For instance, in animals, the proliferation, survival, and functioning of cells are tightly controlled resulting in truly remarkable organisms from annelids and mollusks to amphibians and mammals. Some of the somatic cells retain their capacity to proliferate, others differentiate and lose proliferative capacity. Some cells survive for the entirety of the organism's life, others are destined for rather short lives. For instance, the cells of human epidermis may turnover completely in 40–56 days (even wider variation when accounting for age) and for mice in 8–10 days (Halprin, 1972). Eventually all somatic cells die with the organism. While alive, all cells function as a highly cooperative unit. Selection acting at the multicellular organism level ensures that cells perform as “choreographed” members that send and respond to signals to maintain a “team optimum,” and ultimately contributing to the survival and proliferation of the whole organism.

Unlike normal cells, cancer cells “do reply, do reason why, and do not want to die.” Cancer cells originate from and live in a world where the actions of normal cells are vital to their survival and proliferation; yet, they have broken the compact. They have traits and tools to manipulate normal cells into providing resources, habitats, and protection, and they do so (Sahai et al., 2020; Shiga et al., 2015; Tao et al., 2017). In developing from normal somatic cells, cancer cells take on selfish characteristics that evolve to increase their own individual fitness (survival and proliferation) irrespective of the effect these activities have on other cells or the multicellular group (Gatenby et al., 2020). The proliferation of cancer cells, and their interactions with each other and with normal cells result in complex tumors with emergent properties of well‐organized systems. As cancer cells diversify to fill different ecological niches, the community of cancer cells may manifest cooperation both within and between distinct cancer clones (Capp et al., 2021; Egeblad et al., 2011; Grunewald et al., 2011; Hausser et al., 2019; Heppner, 1989, 1993; Ramón y Cajal et al., 2017). However, the types and eco‐evolutionary drivers of these cooperative interactions are not always clear. Specifically, how is it that cancer cells who increase their individual fitness by exploiting the benefits of cooperation (group optimum) between normal cells then go on to engage in cooperative behaviors (bestow benefits while incurring fitness costs) with other cancer cells?

Darwin puzzled over how cooperation could be the result of natural selection. He, as many subsequently, theorized that individuals that cooperate may prevail in the struggle for existence over those that do not. This generally takes on three very broad (overlapping) categories. In the first, individuals interact nonrandomly so that cooperators are more likely to encounter other cooperators than expected by chance (e.g., kin selection) (Ale et al., 2013; Hamilton, 1964c; Queller, 1992). In the second, individuals come to recognize cooperative individuals from strangers or noncooperatives, and subsequently make their beneficence contingent on what they know of the other (e.g., reciprocal altruism) (Axelrod, 1981; Nowak & Sigmund, 1993; Trivers, 1971). In the third, an individual directly benefits from their action but, incidentally, so do their neighbors (e.g., public goods) (Archetti & Scheuring, 2011; Giraldeau & Caraco, 2018; Hauert et al., 2006). Which strategies are cancer more likely to take advantage of?

**FIGURE 1:**
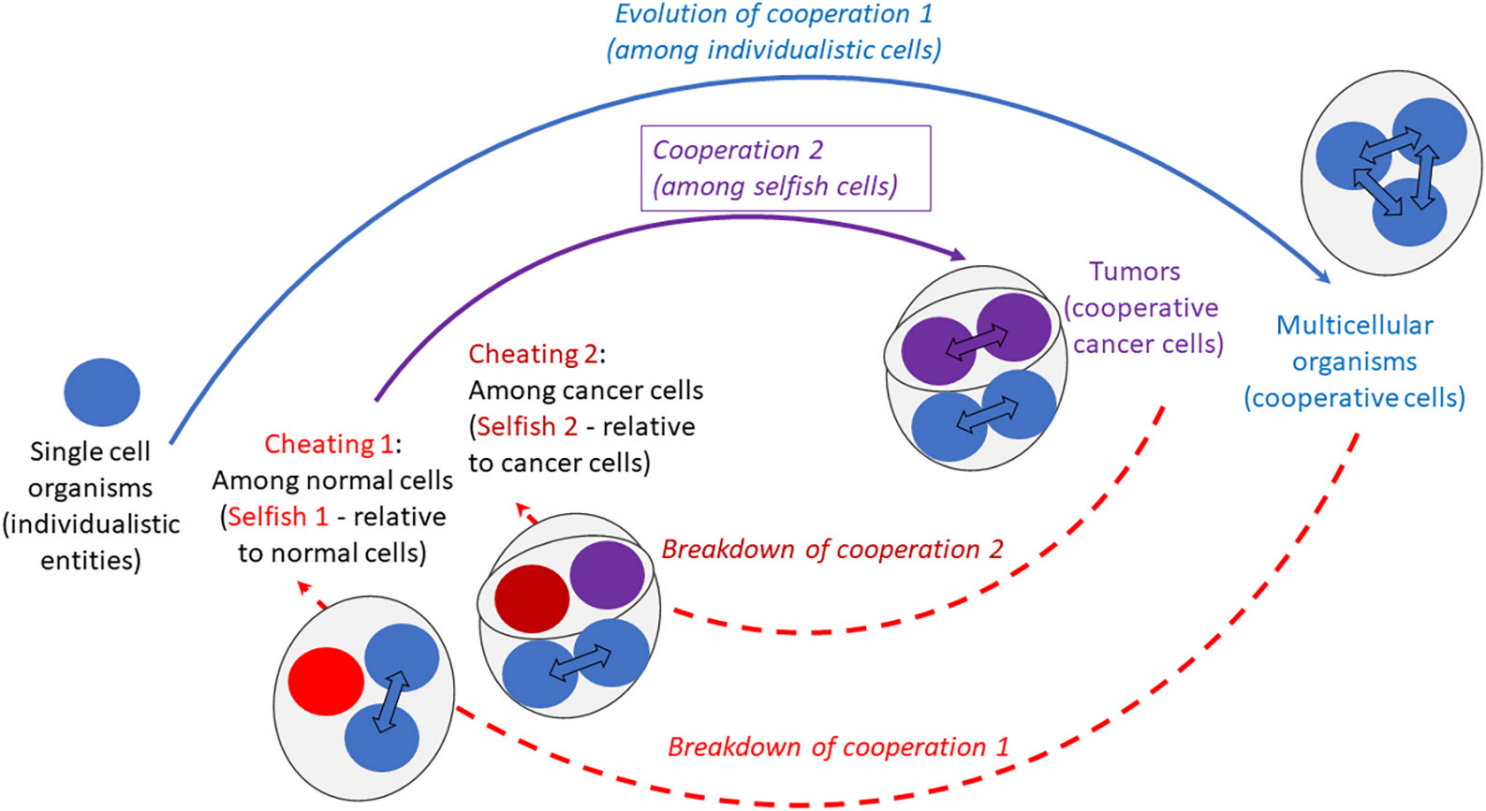
Cycles of cooperation and cheating during the evolution of multicellular organisms and tumor progression. Single‐celled individuals (which can be envisioned as individualistic entities) have engaged in cooperative interactions during the evolution of multicellularity, in which cells cooperate to increase the fitness of the multicellular organism. The evolution of this first cooperative behavior involved new cooperation genes or the co‐option of preexisting genes that served roles at the individual level. However, cheating among cells of a multicellular organism can occur, which can result in the breakdown of multicellularity and the emergence of selfish cells (relative to normal cells). These selfish cells can, nevertheless, engage in cooperative interactions with each other (i.e., a second cooperation cycle), in response to new selective forces and employing mechanisms that are not well understood. Yet, at least theoretically, cheating among such cooperative cancer cells could also occur resulting in selfish cancer cells (relative to the cooperative cancer cells) and the breakdown of cooperation among cancer cells.

In multicellular organisms, it is traditionally accepted that cancer cells develop from normal somatic cells that “cheat.” That is, they lose their typical cooperative behaviors and express selfish characteristics, in the sense that they evolve to maximize their own fitness at the expense of adjacent cells, which are now competitors (i.e., they renounce involvement in cooperative behaviors to increase their own individual fitness by reaping the benefits of cooperation among normal cells without bearing the costs of cooperating themselves) (Gatenby et al., 2020) (Box 1) (Figure 1). Yet, evidence also suggests that solid tumors are complex and well‐organized systems with functional compartments and apparent division of labor, made of a complex consortium of malignant cells that appear to cooperate with each other within individual clonal populations or with cells from distinct clones (Capp et al., 2021; Egeblad et al., 2011; Grunewald et al., 2011; Hausser et al., 2019; Heppner, 1989, 1993; Ramón y Cajal et al., 2017) (Box 2). However, why and how rogue cells that in the context of the healthy tissue act as selfish cheaters would then engage in new cooperative behaviors with other cheaters is not fully understood.

Although cooperative behaviors that evolved in nature are maintained across generations, cooperation among cancer cells within a multicellular individual must emerge de novo in each multicellular individual since, with the exception of transmissible cancers (see (Dujon et al., 2020)), cancer cell populations go extinct with the death of the host (Arnal et al., 2015). Such cooperative interactions can either represent the evolution of new cooperative traits (through mutations) or reflect the expression or co‐option of normal cooperative traits in a different context. In both cases, these cooperative behaviors (altruistic or mutually beneficial) will only emerge under selective pressures and processes favoring interactions that benefit cooperative malignant cells, clones, or tumors over individualistic cancer cells.

BOX 2 **Cooperation in cancer.**

Cooperative behaviors have been implicated in various aspects of cancer (e.g., (Zhou et al., 2017)). Such interactions can (i) be based on either paracrine signaling, cell–cell physical contact, or remodeling of the tumor or tissue microenvironment; (ii) involve related subclones or distant clones; and (iii) be mutualistic, symbiotic, or commensalistic. Below, we provide several previously proposed examples of cooperation affecting the main steps in cancer progression, noting that in some cases these interactions might not fit the narrower/evolutionary definition of cooperation, and/or require additional mechanistic information.

Clonal cooperation affecting tumor growth can involve increased growth factor abundance or activating pro‐proliferative signaling pathways. For instance, glioma cells with mutant EGFR express IL‐6 and/or LIF cytokines, which can activate amplified wild‐type EGFR in neighboring cells, resulting in enhanced proliferation (Inda et al., 2010). Similarly, in a breast cancer context, two subclones with aberrant expression of Wnt1 were necessary for full tumor expansion as one subclone relied on the Wnt1 secreted by the other for growth (Cleary et al., 2014). Furthermore, in another breast cancer model, clone‐specific secretion and reception of factors have been shown to be involved in synergistic growth that can ultimately contribute to tumor progression (Martín‐Pardillos et al., 2019).

Clonal cooperation can also facilitate metastasis, either by causing a phenotypic switch or through microenvironment remodeling. One example of the former mechanism involves poorly metastatic melanoma cells that can uptake tumor antigen‐containing exosomes released by a highly metastatic clone resulting in their own expression of the antigen and increased metastatic potential (Hao et al., 2006). Similarly, breast cancer cell lines could transfer their metastatic potential via secretion of miR‐200‐containing extracellular vesicles (note that these vesicles could enter the circulation and potentially influence distant cancer cells) (Le et al., 2014). Phenotypic switch can also be induced by paracrine factors. For example, in a prostate cancer cell model it was shown that noncancer stem cell (CSC) subpopulations secreted a matricellular protein that induced the invasiveness of a CSC‐enriched subpopulation, leading to enhanced metastasis in lungs (Mateo et al., 2014).

Examples of cooperativity between EMT (epithelial‐to‐mesenchymal transition) and non‐EMT cells that do not require paracrine signaling (but rather microenvironment modeling) have also been reported. For instance, EMT cells can degrade the surrounding matrix to lead the way of invasion and intravasation, which allows the non‐EMT cells to enter the blood stream and establish colonies in the secondary sites (Tsuji et al., 2008). Thus, EMT cells can enhance metastatic properties in cells without causing them to undergo EMT (Neelakantan et al., 2017), or only transiently undergo an EMT (Celià‐Terrassa et al., 2012).

During such collective migration, “guiding cells” that underwent EMT can create migration tracks (signaled by integrin molecules released by the migrating cells during their rear detachment) (Celià‐Terrassa et al., 2012). Similarly, inherently invasive cells exhibiting high protease activity can deposit ECM leading to co‐invasion of poorly invasive cells without the latter undergoing a phenotype switch (instead, the inherently invasive cells switched their invasion pattern). In these cases, the cells with low invasiveness passively benefit from the microenvironmental remodeling ability of the highly invasive cells (Chapman et al., 2014). In other cases, remodeling can involve paracrine signaling and synergistic interactions. For instance, in a rat mammary carcinoma cell line with two stable subtypes, collagenase could only be sufficiently secreted when both cellular types were present. Specifically, a soluble factor released by one subtype induces collagenase secretion by the other (Lyons et al., 1989).

Inherently invasive cells can co‐invade with subpopulations of poorly invasive cells, a phenomenon known as “cooperative invasion.” For instance, melanoma cells with divergent invasive capabilities can interact symbiotically, whereby the underlying cell–cell communications produce reciprocal effects on the individual subpopulations (Chapman et al., 2014). Similarly, in a murine model of small cell lung cancer crosstalk between nonmetastatic neuroendocrine small cells (NE) and mesenchymal large cells (non‐NE) allowed both clones to metastasize, while neither NE nor non‐NE cells formed metastases on their own (Calbo et al., 2011).

Although coexisting heterogeneous tumor populations can support each other via symbiotic type of relationships (Cleary et al., 2014; Inda et al., 2010; Wu et al., 2010), commensalistic interactions are also possible. For instance, in a patient‐derived ovarian clear cell carcinoma model, transient cooperative inter‐clonal interactions that promote metastasis of one clonal population without benefiting the other have been reported (Naffar‐Abu Amara et al., 2020).

The dispersal step can also involve cooperation, through heterotypic or homotypic interactions. In melanomas, proliferative (PRO) and invasive (INV) cells form spatially structured heterotypic clusters and cooperate in the seeding of metastasis. INV cells adhere tightly to each other and form clusters with a rim of PRO cells; during the extravasation step, clusters rearrange with INV cells acting as leader cells (Naffar‐Abu Amara et al., 2020). Collective dispersal can also involve homotypic adhesive interactions between circulating cancer cells in the vasculature or at the site of primary attachment to the endothelium (Glinsky, 2006).

Lastly, cooperative interactions can also affect resistance to treatment, through either paracrine signaling or direct cell–cell connections. For example, cetuximab‐resistant colorectal cancer cells can increase resistance of surrounding sensitive cells in a paracrine manner, through secreting TGF‐beta and amphiregulin (Hobor et al., 2014). On the contrary, astrocytoma cells can interconnect by extending ultra‐long microtubes that allow for multicellular communication through gap junctions, which is critical for invasion and proliferation in the brain. Following damage, new microtubes are extended to the dead cells, allowing interconnected astrocytoma cells to exhibit enhanced radiotherapy resistance (Osswald et al., 2015).

However, as in all cooperative groups, cheater cells within populations of cooperative cancer cells are still expected to occur and take advantage of the benefits produced by the cooperative malignant cells (e.g., in the context of public goods such as angiogenic factors (Archetti & Pienta, 2019); see also (Di Sun et al., 2022; Nagy et al., 2007) e.g., concerning hyper‐tumors) (Figure 1). Such exploitative clones may even drive the cooperative cancer population to extinction (Marusyk et al., 2014). Nevertheless, solid tumors do reach large sizes and complex organization and functionality, suggesting that once established, cooperative interactions among cancer cells can be maintained. Although conceptually sound, this reasoning does not inform on the subtle evolutionary processes (i.e., factors, selective forces, mechanisms) that are behind the emergence and maintenance of cooperation among selfish malignant cells.

Here, we review and discuss the relevance of the different processes that have been proposed, or could be further explored, to explain the evolution/expression and stability of cooperation among cancer cells, both during the progression of tumors as well as during their dispersal (metastasis). We acknowledge that the term cooperation is often used in different contexts (e.g., altruism, mutualism, reciprocity, group selection), with slightly different meanings in terms of benefits and beneficiary, the underlying mechanisms, or its manifestation (complex adaptive behavior or simple by‐product interaction) (see discussion in (West et al., 2007a)). Here, we are adopting the general definition of cooperation as a social behavior that provides a benefit to another individual and evolved at least partially due to that benefit (West et al., 2007a, 2007b). The evolution of such behaviors is generally explained in terms of benefits. The cooperative act can have either direct fitness benefits that outweigh the costs of performing the behavior (through shared interests in cooperation, enforcing cooperation, direct or indirect reciprocity) or indirect fitness benefits by directing the act toward individuals that carry the cooperative gene (involving kin discrimination, limited dispersal or “greenbeard” mechanisms)—though both direct and indirect benefits can also occur (see discussion and figure 1 in (West et al., 2007a)). The first category of explanations is generally associated with mutually beneficial cooperation (which is often used synonymous to mutualism; but see discussion below), whereas the second one refers to costly/altruistic cooperation. Also, note that under this definition, fortuitous mutual benefits such as feeding upon each other's waste products are not considered cooperative behaviors, although in some cases such interactions can be referred to as cooperative (since they benefit one or both interacting partners) and can evolve into cooperative behaviors (see (West et al., 2007a)).

First, we discuss the previously proposed “by‐product mutualism” hypothesis for the evolution of cooperation in cancer (i.e., cooperation among distinct clones through exchangeable resources and capabilities) (Axelrod et al., 2006). Then, we explore the potential relevance to cancer of the main general mechanisms traditionally invoked to explain the evolution of cooperation in nature (kin selection, direct reciprocity, indirect reciprocity, network reciprocity, and group selection; as discussed in (Nowak, 2006)). We also examine the relevance for cooperation in cancer of a new potential contributor, the “greenbeard effect,” in which cooperators can recognize each other via specific phenotypic “markers” (Jansen & Van Baalen, 2006; Riolo et al., 2001; Traulsen & Claussen, 2004). Finally, because malignant cells have access to the vast toolkit of multicellular capacities in the host genome, we propose that they co‐opt/express for tumorigenesis existing adaptations involved in the cooperative activities of normal cells. Our goal is to (i) explore some particular aspects of cancer that might not conform to the usual “rules” of cooperation, especially in terms of the underlying mechanisms, and (ii) highlight the impact of these peculiarities on cancer progression and treatment, noting that in many cases we need additional information to fully characterize these processes. Ultimately, understanding the various cooperative interactions that directly or indirectly increase cancer cells' fitness (regardless of the category they might fit in) should provide new strategies to slow down cancer progression. Overall, we hope this Perspective will stimulate further research that can have therapeutic relevance.

## 2 THE ‘BY‐PRODUCT MUTUALISM’ HYPOTHESIS

The most common explanations for the presence of cooperative interactions among tumor cells fit the “by‐product mutualism” hypothesis proposed by Axelrod et al. (Axelrod et al., 2006). In this framework, partially or fully transformed subclones would exchange diffusible factors associated with their routine activities, which might result in benefits that neither could access alone. Axelrod et al. (Axelrod et al., 2006) argued that cooperation through unidirectional shared benefits (which Axelrod et al. consider as commensalism) or by‐product mutualism would provide some explanations to several observations that have been made about solid tumors, including cooperation between surrounding stroma and the tumor. Among possible examples, the authors discussed the secretion of the vascular endothelial growth factor (VEGF) that triggers new blood vessels within the tumor (neo‐angiogenesis), whereby the diffused oxygen and nutrients would also benefit cells that did not secrete VEGF (unidirectional shared benefit); and the production and exchange of two different growth factors (by‐product mutualism; cross‐feeding) resulting in proliferative capabilities that neither of the subclones would attain on its own (see Box 2 for additional examples). Axelrod et al. (Axelrod et al., 2006) argued that cooperation involving such by‐product mutualistic interactions can evolve more easily because, in contrast to cooperation based on relatedness or reciprocity, it does not require contingent action. Also, this form of cooperation is less susceptible to free riders/cheaters since the activity/product that helps the others is a costless effect/by‐product of a trait that increases the actor's own fitness (Axelrod et al., 2006).

It should be noted that, as in Axelrod et al. (Axelrod et al., 2006), mutualism is often used interchangeably with mutually beneficial cooperation, due to its positive effect on the direct fitness of both partners. However, mutually beneficial cooperation implies a single behavior that affects both the actor and recipient, whereas mutualism describes the effects that each partner has on the other—as between species (see discussion in Ref. (West et al., 2007a)). In the context of Axelrod et al.'s framework, cancer cells' interactions more closely approximate the latter meaning—that is, between distinct species. This is also consistent with the fact that in late stages of carcinogenesis, interacting clones are often genetically and phenotypically very different to the point that some considers them different species (Capp et al., 2021).

Finally, the interactions envisioned in the “by‐product mutualism” hypothesis do not require the evolution of specific cooperative traits/adaptations. Different clones could enhance each other's fitness simply as a consequence of each maximizing their own fitness. Said differently, within a tumor, selection will favor adaptations that increase the fitness of each clone in its local microenvironment, which will also include all of the factors associated with the presence of other clones and cell types. Because within close spatial contexts all clones are under the same selective pressures, at the end there may appear to be selection for the evolution of cooperative interactions, while this is just a by‐product of individual adaptations to the local environment. Below, we discuss and explore other potential mechanisms that can explain the evolution and maintenance of cooperation among cancer cells.

## 3 WHICH EVOLUTIONARY MECHANISMS CAN UNDERLIE COOPERATION IN CANCER?

**FIGURE 2:**
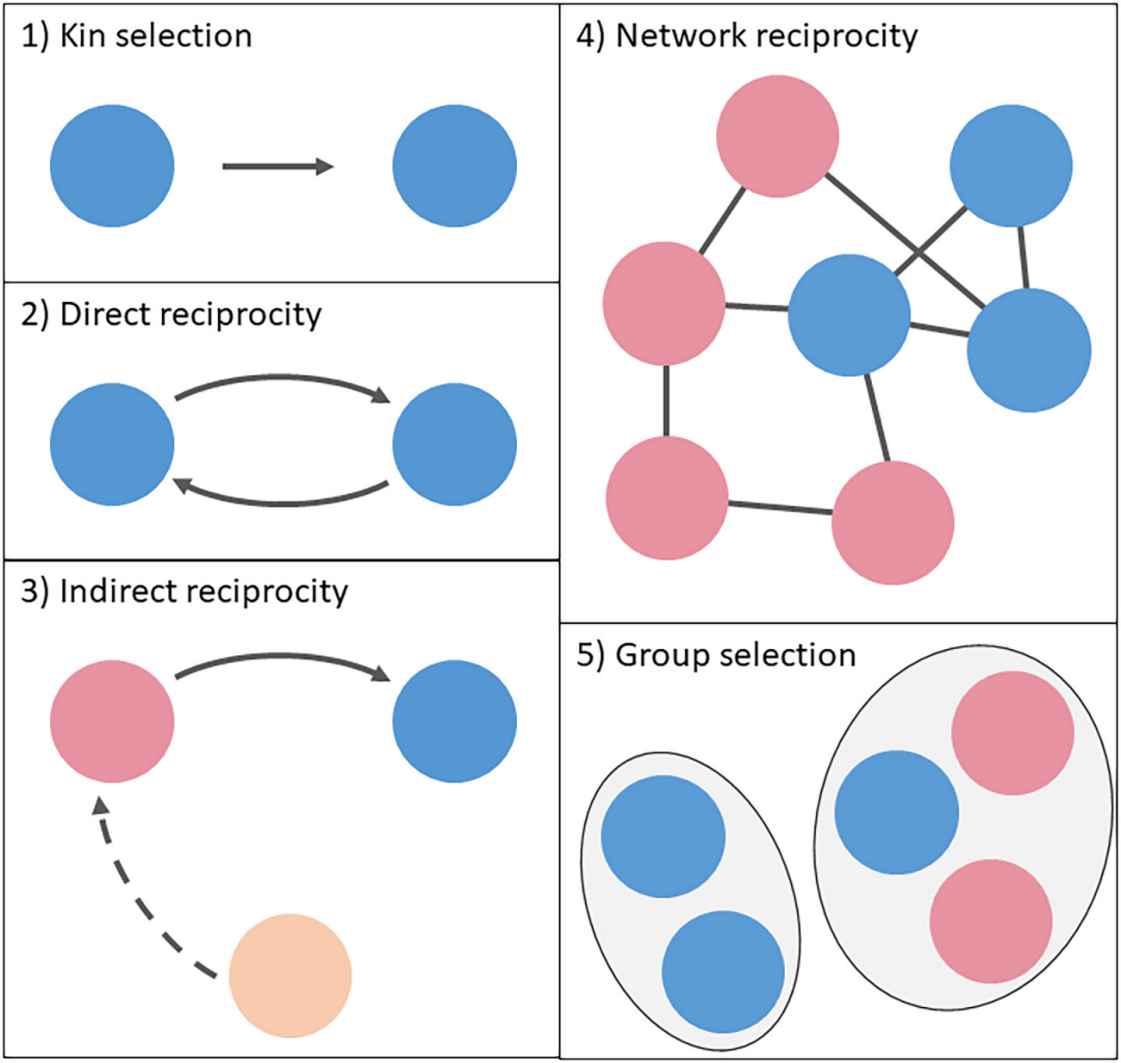
Five main mechanisms involved in the evolution of cooperation. (1) Kin selection requires the donor and the recipient be genetically related. (2) Direct reciprocity operates if there are repeated encounters between the same two individuals. (3) Indirect reciprocity is based on reputation; an individual who helps is more likely to receive help in return. (4) In network reciprocity, clusters of cooperators (in blue) outperform defectors (in red). (5) Group selection can occur if competition is not only between individuals but also between groups, such that groups of cooperators (in blue) outcompete groups containing defectors (in red) (figure modified from (Nowak, 2006)). Colors denote relatedness (same color = related; different colors = unrelated).

Various mechanisms have been proposed to be involved in the evolution and maintenance/enforcement of different forms of cooperation (see discussion in Ref. (Riolo et al., 2001; West et al., 2007a; West et al., 2007b)). Here, we focus on the five main mechanisms described by Nowak (Nowak, 2006) (Figure 2) and discuss them in the context of cancer; that is, which (and under what conditions) such mechanisms might apply or be relevant to cooperation in cancer. For other frameworks that apply evolutionary game theory to the development and maintenance of cooperation in cancer (including the nonlinearity of the benefit of public goods and the role of space and population size), we direct the readers to other references (e.g. (Coggan & Page, 2022)).

### 3.1 Kin selection

Kin selection is a mechanism that can explain the evolution of costly/altruistic cooperation. According to Hamilton (Hamilton, 1963, 1964a, 1964b), costly cooperation can evolve if genes encoding cooperative traits enhance the average fitness of relatives. This is because cooperative individuals, albeit indirectly, can still pass on their genes to the next generation (i.e., indirect fitness benefits; inclusive fitness). While kin selection is generally understood in terms of genealogical relatedness, a broader definition is also often used to include relatedness at the cooperative locus. As predicted by this inclusive‐fitness framework, there are many examples, from microorganisms to vertebrates and even plants, of positive relationships between relatedness and the expression level of cooperative behaviors (Bawa, 1842; Hasegawa & Kutsukake, 2019; Kay et al., 2019; Simonet & McNally, 2021). However, studies linking tumorigenesis and kin selection are scarce and not recent (Orlove, 1977), and it is still debated whether cooperative malignant cell behaviors may evolve under kin selection (Arnal et al., 2015).

Because cancer cells in a solid tumor are most likely clonal derivatives of a single (epi)mutated cell, they are genetically related. Nevertheless, despite this relatedness, cancer cells are far from being identical: as the result of genetic or epigenetic instability, they can display extensive variation (including genetic, cytogenetic, and epigenetic) and phenotypic diversity (i.e., intratumor heterogeneity) (Marusyk & Polyak, 2010). The diversification of subclones through time (Gerlinger et al., 2012) is likely to decrease the relatedness between cells in a way that is proportional to their physical separation within the tumor. In a theoretical paper, Xavier and Chang (Xavier & Chang, 2015) explored a scenario of cooperation among cancer cells where cooperators secrete a growth factor that is costly to synthesize. In accordance with the kin selection theory, they found that cooperation is favored when cancer cells from the same lineage are in close proximity. A decrease in physical proximity and thus relatedness is likely to favor the emergence of cheaters and the collapse of cooperation networks (Figure 1). However, in cooperative groups based on public goods, negative frequency‐dependent selection can prevent the extinction of cooperators (Archetti et al., 2015).

Instead of promoting cooperation, kin selection may also favor, in certain circumstances, the evolution of dispersal as a way to reduce the likelihood of kin competition (Bitume et al., 2013; Ronce, 2007). Limited dispersal is indeed likely to favor spatial proximity between related cells and can increase, especially when resources are scarce, kin competition, which can negate the benefits of cooperation among related cells. Jacob et al. (Jacob et al., 2016) suggested that cooperative individuals should avoid kin competition by dispersing long distances, but also maintain the benefits of cooperation by dispersing in small groups of related individuals. Interestingly, the dispersal step during the metastatic process often involves clusters of circulating tumor cells (CTCs), composed of up to 100 cells (Aceto et al., 2014; Cheung & Ewald, 2016). Several studies have revealed that, compared with single CTCs, CTC clusters have a higher metastatic potential (Castro‐Giner & Aceto, 2020). But further work is needed to determine the degree of cell relatedness within clusters, and the ways in which cells cooperate during the dispersal, dissemination, and colonization steps (Capp et al., 2021). Overall, it is expected that the relative contribution of kin selection might change during carcinogenesis from being more predominant in early solid tumors due to genealogical relatedness and spatial structure, to being replaced by other mechanisms in later stages.

### 3.2 Direct and indirect reciprocities

Direct and indirect reciprocities are mechanisms of conditional cooperation that can evolve in the absence of relatedness (Clutton‐Brock, 2009; Trivers, 1971). Direct reciprocity refers to situations where there are repeated encounters between the same two protagonists, and their respective behavior (cooperation or defection) toward the other depends on what the other did in prior encounters. In these cases, both partners can provide help, which is less costly to the donor than it is beneficial for the recipient (Nowak, 2006). This tit‐for‐tat game theoretic framework has been studied in the iterated Prisoner's Dilemma (Clutton‐Brock, 2009). Although the role of direct reciprocity remains a debated topic, several examples exist in social birds, mammals (Freidin et al., 2017; Kettler et al., 2021) and microorganisms like bacteria (Smith et al., 2020). It is, however, unlikely that the evolution of animal multicellularity initially relied on, or was subsequently maintained because of, clonal cells engaging in reciprocal cooperation (Aktipis, 2020). Note that, we use the term reciprocal cooperation (and not reciprocal altruism) to emphasize that the interactions are mutually beneficial and provide direct fitness advantages to cooperating partners (see discussion in Ref. (West et al., 2007b)). Therefore, if reciprocity exists among tumor cells, there would be two possibilities to explain its evolution: (i) the re‐activation, during or after the malignant transformation, of an ancestral heritable trait acquired prior to the evolution of multicellularity (i.e., an atavism, see for instance (Lineweaver et al., 2021; Vincent, 2012)), or (ii) the evolution of de novo adaptations (see for instance (Thomas et al., 2017)) rendering malignant cells capable of reciprocal cooperation, which involves the capacity to recognize cooperative cells and to express in return reciprocal cooperation toward these cells. Concerning the second option, we come back to a problem raised by Arnal et al. (Arnal et al., 2015). Because the huge majority of cancers are not transmissible, malignant cells are under selective pressures for their new altered life style for, at best, just a few decades (i.e., hundreds or, at most, a few thousands of cell generations). Thus, despite their rapid evolutionary potential, many complex adaptations involving rounds of mutation and selection (as those observed in unicellular lineages exposed to natural selection over millions of years) are unlikely to occur de novo in malignant cell populations.

Indirect reciprocity requires repeated encounters within a group of individuals, for which a donor does not expect a direct return from the recipients (as with direct reciprocity), but rather from other members of the social group; that is, the donor benefits by eliciting help from observers. Although there is some evidence among animals (e.g., (Akçay et al., 2010; Barta et al., 2011; Bshary & Grutter, 2006; Roberts, 2008; Rutte & Taborsky, 2007)), the evolution of cooperation by indirect reciprocity seems to mostly concern humans through, for instance, promoting reputation building and beneficial morality judgments, or other complex social interactions necessitating elaborated cognitive capacities (Clark et al., 2020; Leimar & Hammerstein, 2001; Milinski et al., 2002; Roberts, 2008). Thus, this mechanism is a priori not transposable in the context of cell–cell cooperation.

### 3.3 Network reciprocity and group selection

The theoretical assumption that in a population every individual interacts equally likely with others is an approximation that is rarely met. In many situations, especially when movement and dispersal are limited, individuals cluster and form spatially structured groups for which a lack of overlap prevents spatial reciprocity to occur, canceling opportunities for cooperative behaviors to evolve through network reciprocity (Farine et al., 2015; Su et al., 1900; West & Gardner, 2010).

However, individuals in a group might engage in cooperative interactions that enhance the success or fitness of the group in terms of group stability, viability, or reproduction (Nowak, 2006). Such interactions have been reported to contribute to tumor growth, metastasis, and therapy resistance (see (Zhou et al., 2017) for examples and references) as well as the enhanced dissemination capabilities of CTC clusters (Campbell et al., 2021). Group phenotypic composition (see Glossary) is likely to influence the properties of the group, which in return affect (positively or negatively) the fitness of individual group members (Farine et al., 2015). While a whole tumor is a group of malignant cells, cell movements are limited so that reciprocal interactions among cells are unlikely to be uniform within the tumor. It is therefore expected that tumors consist of more or less functional clusters of interacting/cooperative malignant cells, which could also be in competition (Capp et al., 2021). If some clusters possess a better functional phenotypic composition than others, it is predicted that the most efficient ones could invade the tumor, and ultimately becoming the tumor itself. More research is needed to explore the extent to which tumor dynamics are influenced on the one hand by cooperation between cells and on the other hand by competition between functional clusters of cells. Also, we need more specific information on the spatial organization of tumors and the extent of cell movement in advanced metastatic tumors.

## 4 THE “GREENBEARD EFFECT”—AN UNEXPLORED COOPERATION MECHANISM IN CANCER

In addition to mutualistic interactions involving exchangeable resources/activities that are by‐products of traits that increase individual fitness as well as cooperative behaviors involving genetic relatedness or reciprocity, we discuss another possibility that might favor cooperation between genealogically unrelated clones. Specifically, we explore whether cooperation can also be facilitated by the “greenbeard effect” (Hamilton, 1964b; Jansen & Van Baalen, 2006; West & Gardner, 2010).

### 4.1 What is the “greenbeard effect”?

In its original formulation by Hamilton (Hamilton, 1964b), the concept was used to explain the evolution of altruism even in the absence of genealogical relatedness. In this scenario, an altruism gene can be favored by natural selection if (i) the gene (or a cluster of genes) is associated with a visible phenotype, (ii) the phenotype is used as a “marker” that allows the discrimination between the carriers and noncarriers of the gene, and (iii) the gene induces a preferential altruistic behavior toward other individuals carrying the gene. Because of the benefits resulting from the cooperative behavior, such genes can increase in frequency in the population. Based on this idea, Dawkins (Dawkins, 1976) coined the term “greenbeards,” to support his gene eye's view of evolution.

**FIGURE 3:**
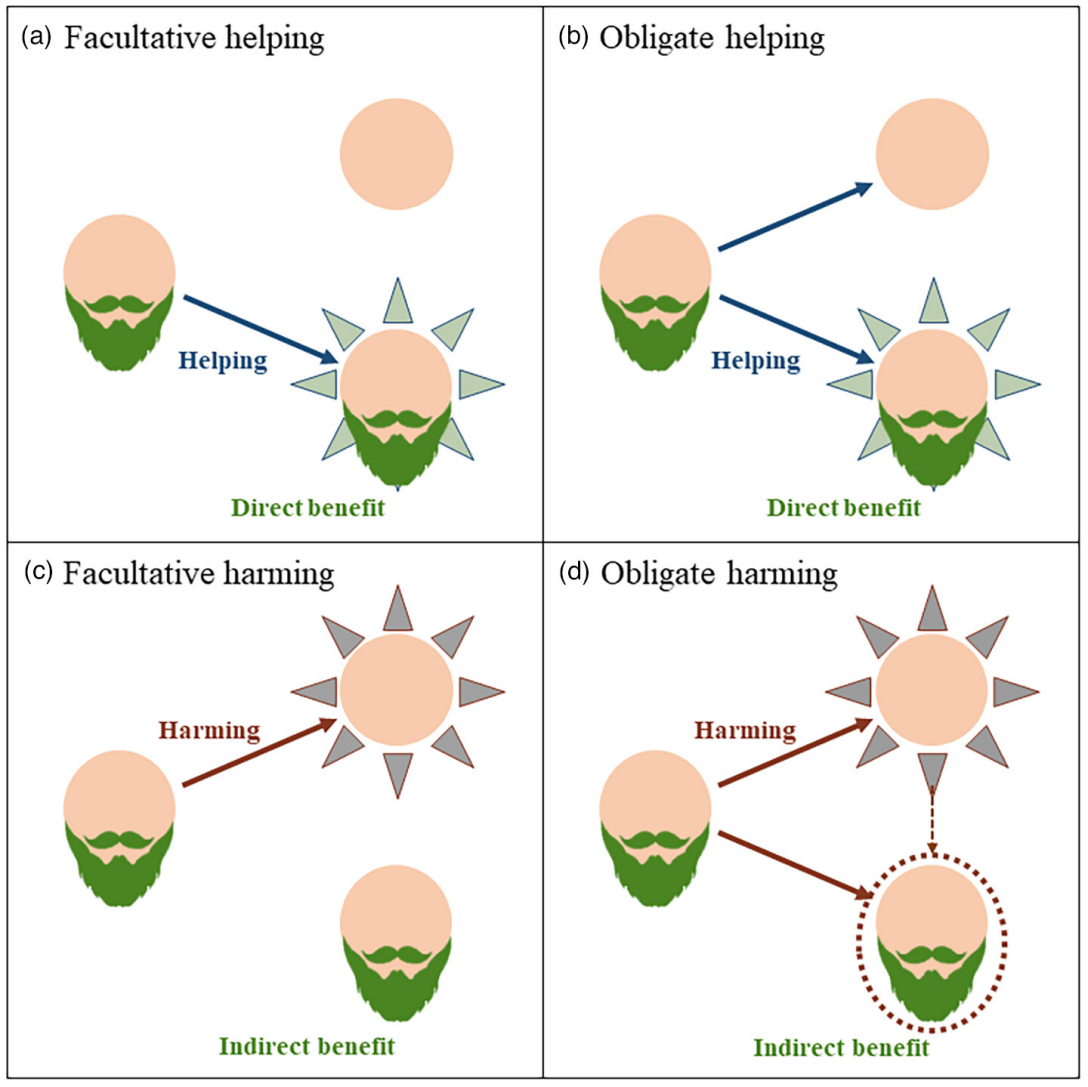
Four categories of greenbeard effects (green and red arrows surrounding the recipient denote helping and harming effects, respectively). (a) Facultative‐helping greenbeard: the greenbeard actor adjusts its optional helping behavior to help only those individuals who are also carriers of the greenbeard marker/gene. (b) Obligate‐helping greenbeard: the greenbeard actor expresses the help behavior toward all individuals, but only carriers of the greenbeard marker/gene can benefit from the help. (c) Facultative‐harming greenbeard: the greenbeard actor optionally adjusts its behavior to inflict harm only on individuals who do not carry the greenbeard marker/gene. (d) Obligate‐harming greenbeard: the greenbeard actor expresses the harmful behavior toward all individuals, but carriers of the greenbeard marker/gene are not affected. In (a) and (b), the benefits of cooperation are direct, while in (c) and (d) they are indirect.

More recently, the concept of greenbeard effect has been extended to include interactions that involve harming of social partners that do not carry the greenbeard gene. This type of interaction is a specific case of spite behavior in which the harm is directed to unbearded (either relative or nonrelative). Notably, harming a noncarrier can benefit greenbeard carriers, and thus, indirectly, contribute a cooperative benefit (i.e., spite can be thought as “altruism towards the secondary recipients” (West & Gardner, 2010)). Furthermore, based on the type (helping or harming) and the facultative or obligate expression of the behavior, four categories of greenbeard effects have been defined: facultative helping, obligate helping, facultative harming, and obligate harming (Gardner & West, 2010) (Figure 3). With the exception of facultative helping, greenbeards are expected to be selected against at low frequencies and only favored once they reached a certain frequency. Population structure (such as in asexual microbial populations; but also in tumors) can help achieve increased frequency by maintaining greenbearded individuals together (Gardner & West, 2010). Also, from a theoretical perspective, spite is thought to be plausible when there is (i) large variation in relatedness among competitors, (ii) kin discrimination, allowing the harming to be directed to nonrelatives, and (iii) strong local competition such that eliminating nonrelative provides strong benefits to relatives (West & Gardner, 2010). Notably, all these conditions are likely to be met in tumors.

### 4.2 Can greenbeard effects play a role in cancer?

Although scarce, current empirical evidence of greenbeard effects has been reported in a diversity of species, including yeast (Smukalla et al., 2008), slime molds (Queller et al., 2003), and fire ants (Keller & Ross, 1998) (see BOX 3 and discussion in (Gardner & West, 2010)). Here, we explore the possibility that greenbeard effects can be involved in cooperative interactions in cancer. We provide potential examples and contexts in which this unexplored mechanism may be relevant to promoting and maintaining cooperation among cancer cells. Furthermore, situations in which greenbeard effects involve harming to nonbearded (and indirect benefits to the bearded) can also be envisioned. Such behaviors might be based on co‐opting the normal cell competition mechanisms in healthy tissues whereby fit cells induce apoptosis in less fit cells (see for instance (Bowling et al., 2019; Krotenberg Garcia et al., 2021)). Indeed, cell competition has been recognized as being relevant to tumor progression in the sense of cancer cells eliminating both surrounding healthy cells as well as less fit cancer clones within a tumor, including by inducing apoptosis (see figure 4 and examples in (Bowling et al., 2019)). However, these interactions have not been interpreted in the context of harming greenbeards promoting indirect cooperation (Figure 3). While the exact mechanisms that allow cells to recognize less fit cells are not well understood, the “fit state” of cells can be considered a phenotypic marker that allows cells to direct harming to cancer or healthy cells that do not express the "marker" (i.e., less fit cells).

BOX 3 **Greenbeard effects in microorganisms.**

Although kin selection is the most accepted explanation for the evolution of altruistic behaviors, these phenomena do not necessarily implicate genealogical relatedness. In a broad definition of kin selection, the individuals sharing the gene of interest might share or not a close genealogical ancestry. In this more general framework, relatedness and genetic similarity are only considered at a particular locus (West et al., 2007a), regardless of its origin (West et al., 2007b). In such circumstances, cooperation can evolve and be stable if this locus exhibits three properties: (1) it produces a conspicuous phenotype; (2) this phenotypic “marker” allows the carrier to discriminate between carriers and noncarriers of the locus; and (3) it leads the carrier to preferentially interact with other carriers of the locus. This scenario is known as the greenbeard effect (Dawkins, 1976).

While examples of greenbeard effects are rare, several microorganisms such as the yeast, *Saccharomyces cerevisiae,* and the slime mold, *Dictyostelium discoideum*, seem to make use of these strategies. For instance, yeast express a multitude of social traits, including biofilm formation, flocculation, plastic adherence, and invasive growth (Hope & Dunham, 2014). These yeast social phenotypes are mediated by interactions between cells that form aggregates through a phenomenon called “flocculation” or “aggregation” (Dranginis et al., 2007). Flocculation is one of the best‐known yeast multicellular growth forms which is classically defined as the reversible calcium‐dependent aggregation of vegetative cells into flocs (Stratford, 1992). It requires proteins, such as adhesins and flocculins, to be expressed on the outer surface of the cell. Flocculins are glycosylphosphatidylinositol‐anchored cell wall proteins encoded by *FLO1*, *FLO5*, *FLO9*, *FLO10*, and *FLO11* (Dranginis et al., 2007). Among these genes, *FLO1* was shown to function as a greenbeard gene during flocculation in liquid medium (Smukalla et al.,  2008). Specifically, *FLO1* ^+^ cells preferentially flocculate with other *FLO1* ^+^ cells, regardless of genetic relatedness across the rest of the genome. This cooperative mechanism protects cells from stressful environments and against cheaters cells (i.e., it excludes *FLO1* ^−^ cells from the flocs). Thus, *FLO1* is a greenbeard gene because it directs cooperation toward other cells carrying the same gene. Another flocculin, Flo11, functioning in cell–cell and cell–surface adhesion is required for many spatially structured social phenotypes in yeast (Bouyx et al., 2021; Oppler et al., 2019) but has not been involved in greenbeard effects *sensu stricto*. Similarly, in *D. discoideum*, the expression of the greenbeard gene, *csaA*, allows carriers to recognize each other, aggregate and form cooperative multicellular fruiting bodies, to the exclusion of noncarriers (Queller et al., 2003).

The greenbeard effects are traditionally considered to involve the gene level because genes are the ultimate units of selection (Dawkins, 1982). However, in the context of cancer, not only genes but also individual cells are considered Darwinian units of evolution (Greaves, 2013), as they possess the required prerequisites (i.e., heritable variation in phenotypic traits that affect fitness (Lewontin, 1970)). Generally, for a greenbeard effect to exist in cancer cell populations, three conditions should be met: (i) genetic or epigenetic components that provide the carrier cells with a phenotypic “marker” (a “green beard”), (ii) cells are able to recognize other cells with the same marker, and (iii) cells sharing the marker preferentially interact with each other in a way that increases their individual or inclusive fitness (i.e., mutually beneficial cooperation or altruism). Although, at least theoretically, these conditions could be met among cancer cells, this possibility has not been explored. Below, we present some potential examples and provide a discussion of the unexplored potential that this mechanism might have for the emergence of cooperative interactions among cancer cells.

### 4.3 Potential examples of greenbeard effects in cancer

A possible illustration of this mechanism is found in the behavior of a particular subset of isolated breast cancer CTCs expressing CD44. CD44 is a hyaluronan receptor known to increase the efficiency of distant metastasis of breast cancer (McFarlane et al., 2015). CTCs expressing CD44 migrate individually to sites of intravasation, but once in blood vessels they cluster through CD44‐mediated intercellular, homophilic protein interactions (Liu et al., 2019). The binding is followed by intracellular CD44–PAK2 interactions and FAK pathway activation leading to enhanced stemness and metastatic propensity. These CD44‐mediated clusters form within the vasculature in the primary tumor, and are then observed in lung metastases, suggesting that CD44 directs the aggregation of individual detached breast tumor cells that enhances metastasis. In contrast, single tumor cells that do not express CD44 die within 48–72 h of detachment. Thus, within the greenbeard framework, CD44 is a phenotypic marker that allows isolated CD44^+^ cells to recognize and preferentially interact with each other through homophilic CD44 interactions; and this interaction increases their survival (as cells in clusters avoid anoikis and immune attacks) and proliferation once they reach the metastatic site.

A similar illustration of this phenomenon has been recently provided, again during lung metastasis of breast cancer (triple‐negative) (Taftaf et al., 2021). In that case, the homophilic interactions between metastatic cells are mediated by ICAM1, and ICAM1‐ICAM1 interactions allow the formation of CTC clusters in circulation. Depletion of ICAM1 avoids this cluster formation and lung colonization. Of note, the expression of ICAM1 is increased by 200‐fold in lung metastases compared to the primary site, suggesting strong selection for high ICAM1 during metastatic spread. Whether the expression of such greenbeards is restricted to members of individual clones or can be shared by genetically distinct clones remains to be addressed.

Overall, such greenbeard scenarios would ultimately lead to an increase in the frequency of cells expressing the markers. Notably, it has been suggested that the greenbeard effect can act even in the absence of a phenotypic marker, as long as the gene (or cluster of genes) for the social behavior codes for an assortment mechanism that ensures the altruistic act is preferentially directed toward carriers of the gene (West & Gardner, 2010). For instance, when the cooperation gene has a pleiotropic effect on habitat preference such that carriers of the gene are likely to colonize the same habitat (Gardner & West, 2010; Hamilton, 1975). In this context, the colonization step during the metastatic cascade might also involve a greenbeard effect as it has been shown that specific metastatic sites (habitats) select for disseminated cells that share specific metabolic capabilities (Schild et al., 2018). For instance, only cells that can cope with oxidative damage can survive and metastasize in the lung, while cells that can thrive in hypoxic environments are more likely to colonize the liver (Schild et al., 2018). If cooperation genes are linked to this habitat preference, colonizing cells are more likely to cooperate during the formation of secondary tumors.

### 4.4 Cancer‐specific considerations

There is a clear similarity between the examples we discussed and the greenbeard effect proposed in yeast (Box 3), where the expression of the flocculation protein Flo1 allows Flo1‐producing cells to preferentially adhere to each other, which protects them from harmful environments (Smukalla et al., 2008). However, in contrast to natural populations of yeast in which the *FLO1* displays considerable sequence variation, the greenbeard effect associated with CD44 or ICAM1 does not necessarily involve new alleles, as all cells possess the same CD44 or ICAM1 gene (see discussion below).

Indeed, in the original formulation, the phenotypic marker (constitutively expressed) is associated with an allele that is restricted to a fraction of the population; and the expression of the allele allows cooperation to be preferentially directed to its carriers (as a recognition or discrimination mechanism) that also share the linked cooperation gene (mediated by linkage disequilibrium) (West & Gardner, 2010). However, in populations of cancer cells, phenotypic heterogeneity (stable or transitory) can also be the result of changes in gene expression. Such changes can affect the *expression* of greenbeard genes that are in fact shared by all members of the population but not necessarily expressed. Thus, potential markers need not necessarily involve new alleles. They could be normal proteins that are abnormally expressed in a fraction of the population. Their expression could be the result of either epigenetic changes or mutations in their regulatory elements (including transcription factors). The marker can also be facultative (i.e., conditionally expressed; induced by the tumor microenvironment) or obligate (constitutively expressed).

Assuming that the phenotypic marker (i) allows the carrier to preferentially interact with other cells that also express it and (ii) is associated or linked to a cooperative behavior, this greenbeard effect could increase the frequency of the *greenbeard‐expressing* cancer cells. In this scenario, however, selection will act on the *mechanism(s) that induced the expression of the green beard*, rather than on the greenbeard gene itself, since the gene can in fact be carried (but not expressed) by all members of the population. This proposed mechanism represents a new way in which cancer cells can cooperate by taking advantage of normal genes that can act as greenbeards when they are abnormally expressed in a subset of the population, and preferentially direct cooperative interactions among the members of this subset. This mechanism is possible in cancer because of the epigenomic instability and the high rate of mutation as well as the fact that many different mutations can directly or indirectly affect the expression of preexisting normal genes. Paradoxically, cooperation might even ensue among cells that do not use the same genetic mechanism to express the greenbeard gene (i.e., the expression of the phenotypic marker can be due to different mutations or epigenetic changes) as the same phenotype can be associated with distinct genotypes (i.e., convergent evolution). Such a scenario will allow the maintenance of genetic heterogeneity in a tumor, even among cooperative cells expressing the same phenotypic marker.

In addition to (epi)mutations that can affect the expression of the marker and facilitate greenbeard‐mediated cooperation, several other factors might result in the differential expression of the greenbeard gene. For instance, differential splicing results in CD44 being expressed as a series of standard and variant isoforms, with distinct effects on breast cancer progression and metastasis (McFarlane et al., 2015). Furthermore, in isolated cells, including CTCs, whose phenotypic behavior is not coordinated by microenvironmental/tissue constraints anymore, the ancestral cooperation toolkit (including cell–cell adherence) inherited from the normal cells can be stochastically expressed (Capp & Thomas, 2020). If the expression of a marker in several cells allows them to recognize and interact with each other (as for the example of CD44^+^ or ICAM1^+^ cells in the vascular system), and if this interaction increases their fitness, a greenbeard effect would occur, leading to an increase in the frequency and level of expression of the phenotypic marker without the need for mutation. Here, the factor inducing the expression of the greenbeard gene would not be a mutation or an epimutation, but rather the loss of the initial coordination of gene expression in the healthy tissue linked to a tissue disruption (Capp, 2005) that causes unicellular‐like behavior (Capp & Thomas, 2020). Finally, CD44 and ICAM1 can be upregulated by environmental factors such as cytokines and growth factors (Chen et al., 2018; Figenschau et al., 2018). Thus, besides epimutations and stochastic expression, the green beard (over)expression could be favored under certain environmental conditions. In the framework of a nongenetic origin of the presence of the green beard in some cells, this would mean that the collective behavior would be more frequent in specific environments, creating a window for intervention at the microenvironmental level to limit such behavior.

### 4.5 Implications of greenbeard effects to cancer progression

The novelty of this greenbeard hypothesis in cancer also offers new perspectives on intratumoral heterogeneity (ITH). Indeed, greenbeard effects can be relevant to understanding the group phenotypic composition (GPC) of tumors (Capp et al., 2021), because groups could be made up of cells with cooperative behaviors whose assortment was driven by expression of similar phenotypic markers, not necessarily based on genetic relatedness. This would structure the tumor and result in ITH. Such effects would be essential in tumors because they would ensure (1) cooperation between cancer cells (in the sense of increasing their own direct or indirect fitness) which were initially selfish due to the loss of coordination at the tissue level, and (2) protection against the adversity of the tumor microenvironment in which they must survive and grow. A possible limitation of the relevance of greenbeards dynamics in the context of tumorigenesis could be the lack of significant cell movement within the tumor, implying that cells interact mostly with neighboring cells. However, in metastatic tumors and during invasion and dispersal, these constraints are released.

Greenbeard effects are generally considered to be of minor relevance to the evolution of cooperation because of the danger of “falsebeard” cheaters. However, in microorganisms—which have a simple genotype/phenotype map, the decoupling of the greenbeard gene from the cooperative behavior is thought to be less likely (Gardner & West, 2010). Provided that some of the greenbeards that cancer cells might express are strongly linked to the cooperative behavior, cooperation can also be stable. For instance, similar to the yeast example (and a similar example in slime molds; (Queller et al., 2003) Box 3), cells not expressing the CD44 or ICAM1 marker (i.e., avoiding the cost of cooperation) will not be able to interact with cooperators expressing CD44 or ICAM1, respectively, and thus will not benefit from the cooperative behavior expressed by the cooperators.

Finally, an intriguing possibility, although speculative at the moment, would be that greenbeards in malignant cells are a legacy from oncogenic viruses. Many cancers have an infectious causation (Ewald, 2009; Ewald & Swain Ewald, 2015), and viruses, including oncogenic ones (e.g., herpes viruses), are also involved in various interactions both within and between infected cells (Díaz‐Muñoz et al., 2017), promoting for instance a social behavior in the infected cells (see for instance (Ejercito et al., 1968; Keller et al., 1970; Ruyechan et al., 1979)). It is unclear at the moment if the social behavior expressed within clusters of infected cells is governed by greenbeard processes, but if this were the case, one could speculate that some proximate causes of this dynamic are subsequently adaptively maintained by cancer cells during tumorigenesis.

Overall, further research is needed to explore the possibility that greenbeards play an important role in the expression of cooperative behaviors among malignant cells. For instance, it would be useful to design protocols to study the interaction between malignant cells from different primary tumors within the same patient (e.g., (Vogt et al., 2017))—which will ensure that cancer cells are genealogically distinct but share histocompatibility, or between primary tumors from the same organ of the same or different patients—which will address whether the phenotypic marker can be specific to, and act toward cancer cells sharing the tissue of origin, regardless of genealogy and/or histocompatibility. These experiments would provide direct evidence for the existence of greenbeard effects and their mechanistic basis. Understanding such mechanisms might explain aspects of cancer biology in contexts that are otherwise difficult to reconcile with the existence of cooperative interactions.

## 5 THE PARADOX OF COOPERATION AMONG CANCER CELLS

As discussed in the Introduction, the idea of cheater cells cooperating seems paradoxical at first. How/why would selfish cheaters—which by definition are noncooperators, “learn” to cooperate with each other? And why are such apparently new cooperative behaviors so successful, in terms of both the multitude of benefits they can provide and their stability in the face of new types of cheaters? In the previous sections we briefly introduced the main classical evolutionary mechanisms that have been proposed to explain the evolution of cooperation in general. We also tried to apply them in the context of cancer in terms of understanding if/how they could be used to explore interactions among cancer cells that can be perceived as cooperative and contribute to cancer progression. Below, we discuss several additional cancer‐specific aspects that need to be considered when attempting to answer these questions.

For instance, there are several distinct types of cooperative interactions that can be expressed in cancers (possibly in the same tumor), depending on genetic relatedness (within a clone or between clones), the type of cooperation (mutually beneficial or costly, within a clone; unidirectional or mutualistic, between clones), or type of benefit (public goods; exchange of goods; division of labor) (see Box 2). The reasons for this mosaicism are also multiple. For example, the clonal nature of tumors could allow for kin selection to operate and favor intra‐clonal cooperation (e.g., public good production), whereas the high genomic instability and mutation rates that result in genetic heterogeneity can allow for inter‐clonal cooperation (e.g., mutualism or inter‐clonal division of labor). Similarly, phenotypic plasticity within a clone allows subclones to express different plastic phenotypes (intra‐clonal division of labor), while the ability of a clone to alter the phenotype of a different clone can allow one clone to recruit another clone into expressing a phenotype that can increase the fitness of both clones (Box 2). Mechanistically, the underlying cause for the ease with each these different cooperative interactions emerge is that in most cases they involve the re‐activation of cooperative traits that are generally expressed during normal development or in other tissues and contexts (e.g., expression of public goods such as growth factors; production of VEGF; phenotypic switching). Selection will nevertheless have to “match” these cooperative traits with the genetic (in terms of relatedness) and microenvironmental context in which they are re‐expressed for cancer cells to benefit from the cooperative interaction.

Furthermore, although cancer cells are generally viewed as a reversal to a single‐cell “selfish”/individualistic life style and therefore subjected to cell‐level selection, this might not necessarily always, or fully, be the case. In addition to reproductive altruism (relative to the germ cells), somatic cells express a variety of cooperative activities (among themselves) that enhance the functionality of the body/soma and ultimately increase the fitness of the multicellular organism. Cancer cells lose some cooperative traits expressed by the normal somatic cells (i.e., control of proliferation, response to inhibitory signals or apoptosis), but not all. For instance, most cells in tumors still express an important cooperative gene/trait set—that is, cell–cell adhesion. Although oncogenic cells detach themselves from the normal cooperative structural framework of tissues (e.g., the basal membrane), once they start dividing, their progeny remain attached through normal cell–cell adhesion molecules (e.g., E‐cadherins) and structures (e.g., desmosomes) to each other, and form a multicellular clonal group in a similar way that clonal multicellularity evolved and develops (i.e., by cells “staying together”). This means that, in fact, in solid tumors cheaters already emerge as cooperative cheater groups within a normal cooperative group.

The fact that cancer cells maintain cell–cell adhesion might contribute to the success of early tumors as cheater groups that continue to enjoy some of the benefits of the ancestral cooperative normal cells they defaulted on. Likewise, new clones in a tumor also arise as new cooperative cheater groups (from another cheater group; i.e., cheater diversification) that might compete or cooperate with the neighboring groups. This fundamental cooperative trait is down‐regulated during the initiation of metastasis as individual cells transition from epithelial‐to‐mesenchymal states (EMT), but presumably is upregulated once the cells resume proliferation at new locations to form new cooperative cheater groups based on the ancestral “staying together” cooperative strategy. Since this step is involved in the dissemination of cancer cells, the acquisition of the single‐celled state might just be a dispersal strategy (not a reversal to cell‐level “selfishness”). Nevertheless, in some instances this cooperative trait can still be maintained, allowing for collective migration and dispersal (i.e., CTC clusters).

Once cells accumulate genetic or epigenetic changes (or intratumoral selection is relaxed, and/or drift is strong) that result in higher levels of clonal diversity, additional intra‐clonal or inter‐clonal cooperative behaviors could be expressed. But such changes are also expected to affect cooperative behaviors; and, as exemplified by cancer cells themselves, cooperation can be threatened by the emergence of cheaters that take advantage of the benefits of cooperation, without paying the costs (Figure 1). Is cooperation among cancer cells susceptible to cheating? And if so, why is cheating not able to drive cancer cooperative groups to extinction? What are the potential mechanisms that might control/suppress cheaters within a cooperative cancer clone/group? The answer to these questions might also be as multiple as the types of cooperation that can be expressed in cancer. In addition to the common mechanisms proposed to suppress cheating in microbial populations (André & Godelle, 2005), one possibility is that due to the high rate of mutation, cheating on cheaters might in fact stabilize cooperation, as it has been shown in the bacterium *Pseudomonas aeruginosa* (Özkaya et al., 2018). Alternatively, since cancer cell populations are polymorphic at many traits and are exposed to various microenvironmental conditions, they are likely subjected to multi‐directional interactions under multiple environments. In such multi‐trait/multi‐constraint settings, an individual can behave simultaneously as a cooperator and as a cheater for different traits (see discussion in (Özkaya et al., 2018)). Such scenarios can affect the dynamics between cooperators and cheaters and contribute to the generation of interdependent interactions ultimately having the potential to result in an apparent division of labor (Özkaya et al., 2018). Also, public good‐based cooperative groups are likely to resist cheaters if the cost of producing the public good is low (e.g., (Chapman et al., 2014)), particularly when selection is weak.

Considering how diverse cancer cell populations are, and how these populations can change over time, it is conceivable that all the cooperation mechanisms described above can occur in different tumors (or parts of tumors) and/or contexts, and at different times. We can however expect the likelihood of the different mechanisms for cooperation to be different, spatially, and temporally. Relatively complex types of cooperation may appear relatively easy (in a small number of generations under specific selective pressures) during cancer progression if they are based on the expression (or abnormal activation) of genes and pathways already present in normal cells. In contrast, other cooperation strategies may be less common and only appear under certain conditions. Furthermore, shifts in cooperation strategy may be required at multiple stages of tumorigenesis process (e.g., during angiogenesis, as tumor microenvironment changes, or during metastasis).

## 6 THERAPEUTIC IMPLICATIONS

The vast majority of both current and under development therapies are directed toward killing or suppressing the proliferation of tumor cells. Despite the introduction of highly effective drugs, such as tyrosine kinase inhibitors of EGFR or ALK signaling pathways, our ability to eradicate tumors is limited by ITH in therapy sensitivity, where some tumor cells avoid elimination due to genetic, epigenetic, or microenvironmental differences (Lindsay et al., 2017). Development of complex combination therapy schedules that target multiple mechanisms of persistence and resistance operating within the same tumors is limited by availability of drugs and issues of systemic toxicities. In principle, targeting mechanisms of cooperation, rather than focusing on individual subpopulations might overcome at least some of these limitations by both providing new potential molecular targets and “killing many birds with one stone.”

The acknowledgment that cancer cells engage in cooperative interactions opened up the possibility that, at least in some instances (e.g., in tumors that are not dominated by drift), new therapeutic approaches can be developed to disrupt cancer cell cooperation or facilitate the success of cheaters (Archetti & Pienta, 2019). Targeting cooperative behaviors (especially involving communication and diffusible factors) has also been suggested to be a more effective strategy than the administration of regular antibiotics against bacterial pathogens as such adaptations are thought to evolve slower (André & Godelle, 2005). Several candidates for the disruption of clonal cooperation in cancer have been suggested, including Wnt1, MMPs, and Hedgehog (see (Zhou et al., 2017) for further discussion and limitations). Alternatively, it has been proposed that cooperation can be disrupted (resulting in tumor collapse) through the introduction of engineered cancer defector cells (Archetti, 2013, 2021). Moreover, consideration of cooperation within tumors might allow the development of better approaches regarding the administration of current therapies. For instance, it has been suggested that a growing cooperative tumor should be “hit hard” to avoid changes in its composition that might allow the tumor to withstand the stress (Capp et al., 2021). In the absence of cooperation abilities, cancer cells should not be able to build functional and metastatic tumors, and tumors would probably not result in the major health problems associated with cancer.

Obviously, development of strategies aimed at disrupting cooperation will also face challenges. Most cooperative behaviors expressed by cancer cells are also expressed by normal cells (e.g., cell adhesion molecules, growth factors). Moreover, cooperative behaviors might be mediated by multiple molecular mechanisms, and neoplastic populations might be able to evolve resistance to cooperation targeting strategies by shifting to alternative options within a large repertoire of cooperative behaviors that they inherited from the normal cells. In addition, optimization of strategies directed against disrupting interactions will likely be more complicated compared with more simple direct kill approaches, as predicting behavior of complex dynamic systems requires use of modeling tools and accurate understanding of the underlying dynamics. Despite these challenges, we posit that deciphering mechanisms that underlie biological interactions within subpopulations of tumor cells would not only improve our understanding of basic tumor biology but also increase the arsenal of therapeutic options in an oncologist's quiver, and, potentially, open new opportunities for groundbreaking therapeutic developments.

## 7 CONCLUDING REMARKS

Although cancer cells are genetically and evolutionarily related to the normal/host cells, the emergence of cooperative behaviors among cancer cells is not necessarily analogous to the development of cooperative interactions during the evolution of animals, because the selective pressures, evolutionary mechanisms, and genetic basis are different (Figure 1). For instance, the evolution of cooperation in early multicellular animals is thought to have been driven by the benefits of large size in terms of avoiding predation and/or maintaining an internal homeostatic environment. On the contrary, although the advantages of cooperation for CTC clusters can be partially associated with escaping immune predation (but also avoiding anoikis), cooperation within tumors is likely driven by the need to survive and proliferate in an increasingly deteriorating microenvironment. The factors affecting the likelihood and stability of cooperation are also very different; for instance, genetic relatedness plays less of a role in cancer (at least in advanced tumors), while unidirectional and mutualistic interactions between genetically distinct clones can greatly contribute to the evolutionary success of tumors. In addition, greenbeard effects that allow unrelated individuals to interact and cooperate are likely to facilitate cooperation in cancer, as many of the normal genes can be expressed abnormally in a subset of cells, and the expression of such markers can be induced by a variety of processes. Lastly, the genetic basis is very different between the two levels of cooperation. While cooperation during the evolution of animal multicellularity involved new genes or co‐option of old genes into new pathways, cooperative interactions among cancer cells are likely to mainly involve changes in the expression of existing genes. Also, while some of these changes might be due to genetic or epigenetic mutations, the increased cellular stochasticity observed in cancer cells could also be “exploited” to induce cooperative behaviors that are based on the expression of genes (including genes that can act as greenbeards) that have been selected for during the evolution of cooperation in animals (e.g., cell adhesion, cell communication, cell differentiation). Therefore, a better understanding of the complex rules that govern the emergence of cooperation among selfish malignant cells could have major therapeutic implications.

## Glossary

**Inclusive fitness**: term introduced in 1964 by William Donald Hamilton to measure evolutionary success. Inclusive fitness is defined as an individual’s direct fitness plus an individual’s indirect fitness, where (a) direct fitness is the number of offspring directly produced by an individual, and (b) indirect fitness is defined as the number of related individuals produced, multiplied by the degree of relatedness of those individuals.

**Multilevel selection**: when selection operates simultaneously on several levels of organization at the same time, often favoring cooperation at the group level and exploitation or cheating at the individual level.

**Relative fitness**: a way of measuring the reproductive success of organisms, in which the rate of reproduction of one phenotype or genotype within a population is relative to the maximum rate of reproduction of other phenotypes or genotypes.

**Group Phenotypic Composition**: term introduced by Farine et al. (Farine et al., 2015) to describe any descriptor of the types of phenotypes present within a group (e.g., body size, color, aggressiveness). The heterogeneous composition of groups gives them different properties, which in turn can influence group‐level outcomes (e.g., predation risk, evolvability). Capp et al. (Capp et al., 2021) translated the conceptual framework to tumors, considering descriptors like, for instance, average proliferation rate, degree of genetic or phenotypic heterogeneity, proportion of cells with distinct differentiated states etc., arguing that it can help understand the evolution and clinical progression of cancer.

## Data Availability

Data sharing is not applicable to this article as no new data were created or analyzed in this study.
